# Enhanced Visible Light Active WO_3_ Thin Films Toward Air Purification: Effect of the Synthesis Conditions

**DOI:** 10.3390/ma13163506

**Published:** 2020-08-08

**Authors:** Anna Pancielejko, Marta Rzepnikowska, Adriana Zaleska-Medynska, Justyna Łuczak, Paweł Mazierski

**Affiliations:** 1 Department of Process Engineering and Chemical Technology, Faculty of Chemistry, Gdansk University of Technology, 80-233 Gdansk, Poland; anna.pancielejko@pg.edu.pl (A.P.); justyna.luczak@pg.gda.pl (J.Ł.); 2 Department of Environmental Technology, Faculty of Chemistry, University of Gdansk, 80-308 Gdansk, Poland; marrzepn21@gmail.com (M.R.); adriana.zaleska-medynska@ug.edu.pl (A.Z.-M.)

**Keywords:** WO_3_ nanoflowers, electrochemical anodization, toluene degradation, visible light-induced photocatalysis

## Abstract

Taking our current environmental situation in the world into consideration, people should face growing problems of air and water pollution. Heterogeneous photocatalysis is a highly promising tool to improve both air and water quality through decomposition/mineralization of contaminants directly into harmless CO_2_ and H_2_O under ambient conditions. In this contribution, we focused on the synthesis of self-assembly WO_3_ thin films via an electrochemical approach in the aqueous electrolyte containing fluoride ions toward air purification. The effect of preparation conditions such as applied potential (10–50 V), anodization time (15–120 min), concentration of H_2_SO_4_ (0.5–1.5 M) and NaF (0.1–1.0 wt.%) on the morphology, photocurrent response, and photocatalytic activity addressed to removal of air pollutant in the presence of as-prepared WO_3_ samples were thoroughly examined and presented. The results revealed the growth of nanoplatelets and their gradual transformation into flower-like structures. The oxide layers and platelet thickness of the WO_3_ samples were found to be proportionally related with the synthesis conditions. The photocatalytic ability toward air purification was evaluated by degradation of toluene from air mixture using low-powered LEDs as an irradiation source (λ_max_ = 415 nm). The highest photoactivity was achieved in presence of the sample which possessed a well-ordered, regular shape and repeatable distribution of flower buds (100% of degradation). The results have confirmed that the oxide layer thickness of the anodic WO_3_ significantly affected the photocatalytic activity, which increased with the increasing thickness of WO_3_ (to 1.05 μm) and then had a downward trend. The photocurrent response evidenced that the well-organized sample had the highest ability in photocurrent generation under UV-Vis and Vis irradiation. Finally, a possible growth mechanism of WO_3_ NFs was also discussed.

## 1. Introduction

Air pollution is the major cause of global environmental threats. It contributes to the formation of respiratory diseases, infectious diseases, acute toxic effects, cancers, reproductive disorders, and allergies. The World Health Organization reports that over 4.2 million deaths result from exposure to ambient air pollutants every year [1]. Moreover, air pollutants adversely affect the world, disrupting photosynthesis, transpiration, and respiration. Secondarily, they contaminate water and soil. They increase the acidity of drinking water as well as the contents of heavy metals such as lead, copper, zinc, and cadmium in the water. Acidified water destroys plumbing installations, washing away various toxic substances from them. They cause corrosion of metals and building materials. Air pollutants also have a negative impact on climate change. The main problem which needs to be solved today it is a successful decomposition/mineralization of air contamination. Therefore, an innovative and effective method of removing harmful substances from the environment is sought.

Advanced oxidation processes (AOPs) have been extensively studied to reduce a great variety of pollutants present in various environmental media [2,3,4]. Among them, special attention has been paid to the use of heterogeneous photocatalysis in environmental applications for the remediation of polluted air and water. The photocatalytic effects exerted by redox reactions are caused by photoinduction of electrons (e^−^) from the valence band to the conduction band, leaving positive holes (h^+^) behind in a very short time (femtoseconds) under the influence of UV-Vis irradiation. Several reactive species, including highly reactive hydroxyl radicals (•OH) and superoxide radicals (O_2_^•^^−^), are generated through the reaction of e^−^ and h^+^, which are considered to be involved in the oxidative and reductive reactions in photocatalysis. The generated reactive oxygen species react with adsorbed gas and/or water pollutants, leading to the degradation/mineralization them into CO_2_ and H_2_O [5,6]. Successful photocatalytic air purification relies on the following parameters: (i) photon absorption of semiconductor photocatalysts and (ii) surface properties of photocatalysts, in particular, keeping clean photocatalyst surfaces free from the accumulation of recalcitrant intermediates and products during the processes [7,8].

Currently, the most studied photocatalyst, which holds great potential as a very effective photoinduced photocatalyst, is titanium dioxide (TiO_2_). It is frequently used to decompose organic and inorganic contaminations from water and air due to its good chemical stability, high oxidizability, non-toxicity, and low-cost preparation of nano- and microparticles. However, the main drawback of it is the wide band gap (3.2 eV for anatase), and consequently, only a small fraction of the solar irradiation can be absorbed by this material (~4%) [9,10]. Many studies reported that sufficient doping of cations or anions into TiO_2_ layers to create certain states within the band gap energy may lead to achievement of visible light-driven TiO_2_ photocatalysts [11]. However, those modifications have a number of failures, such as their thermal instability and formation of recombination centers for photoinduced charge carriers, which significantly decrease their photocatalytic abilities [12]. Therefore, design and/or further development of an efficient visible light active photocatalyst is a particularly critical need for air purification.

From a practical point of view, tungsten trioxide (WO_3_) became an alternative photocatalytic material with interesting optical, electrical, and structural properties [13]. In terms of chemical stability and inertness, WO_3_ exhibits the same attributes as TiO_2_. In addition, it possess a lower band gap energy level (between 2.4 and 2.8 eV), stable physicochemical properties, and strong resilience to photocorrosion effects [10,14]. Furthermore, it has been considered a great interest due to its ability to (i) decontaminate polluted water [15,16], (ii) detect hazardous gases [17,18,19], and (iii) convert solar energy [20,21].

Numerous studies reported different synthesis approaches of WO_3_ nanostructures, including sol-gel technique [22,23,24], electrochemical deposition [25], chemical vapor deposition [26,27], electrochemical oxidation [28,29], magnetron sputtering [30,31,32], ion-beam evaporation [33,34], and atomic layer deposition [35], which have been described. Among them, an anodization technique attracts considerable attention due to its low cost and the simplicity of a synthesis route. Furthermore, the possibility of controlling and adjusting a growth process by tuning anodization parameters (such as applied potential, synthesis time, and temperature) as well as electrolyte composition makes this technique more interesting for fabricating metal oxide nanostructures with controllable pore size, good uniformity, and conformability over large areas [36,37,38]. However, several studies have reported that it is still a challenge to obtain well-aligned and uniform anodic WO_3_ nanostructures instead of nanoporous structures [13,39,40,41]. Preparation of porous WO_3_ using galvanostatic anodization of W foil in an oxalic acid electrolyte was first described by Grimes et al. [42]. It was found that the obtained nanoporous tungsten oxide exhibited a more regular surface with smaller pore size in compare with results described previously for other methods. A self-organized nanoporous structure by anodizing W foil in electrolyte composed of 1 M sulfate acid and 0.5 wt.% sodium fluoride was reported by Schmuki et al. [37]. The ability to control the synthesis conditions allowed them to obtain the desired self-ordered oxide structure exhibiting higher photocurrent efficiency than a compact oxide layer. Sadek et al. described the growth process of WO_3_ nanoplatelets during the anodization of tungsten foil in a nitric acid environment at higher temperatures [43]. The combination of the synthesis parameters with high temperature led the authors to obtained relatively thick films of nanoplatelets with potential application in photosensitive devices. A flower-shaped tungsten oxide nanostructure prepared in an acidified electrolyte solution containing fluoride ions was reported by Amal et al. [29,44]. The resultant thin oxide film, with enhanced surface area and thickness, exhibited a higher photocurrent density. Furthermore, the annealing temperature and crystallite of the as-anodized WO_3_ nanostructures were found as critical factors in the water splitting reaction [44]. Despite many reports describing the synthesis approach of WO_3_ nanostructures with the electrochemical method, there is still a lack of knowledge about the effect of synthesis parameters (such as electrolyte composition, applied potential, and time period duration) on the geometrical parameters, optical properties, and photocatalytic properties with photoactivity of the WO_3_ nanoflowers.

Motivated by this, we propose, for the first time, a novel synthesis method of the self-assembly three-dimensional tungsten oxide nanoflowers (3D WO_3_ NFs) thin film with improved optical and photoelectrochemical properties and exhibited enhanced photocatalytic activity in the reaction of toluene degradation under visible irradiation. The objectives of the present study are, therefore: (i) to optimize synthesis conditions of the WO_3_ NFs, (ii) to correlate morphological dimensions and photoactivity, and (iii) to find optimal synthesis conditions to assure that the nanostructures do not decompose under illumination and that they remain stable over time.

## 2. Materials and Methods

### 2.1. Materials

Isopropanol (p.a., POCh. S.A., Gliwice, Poland), acetone, methanol (p.a., P.P.H. STANLAB, Lublin, Poland) and deionized water (DI, with conductivity of 0.05 µS) were used during the sonication process. WO_3_ NFs were synthesized using anodic oxidation of tungsten foil (0.127 mm, 99.9% purity, Sigma-Aldrich, Darmstadt, Germany) in the aqueous electrolyte composed of sulfuric acid solution (96%, p. a., P.P.H. STANLAB, Lublin, Poland) and sodium fluoride (p. a., P.P.H. STANLAB, Lublin, Poland).

### 2.2. Synthesis of WO_3_ Nanoflowers

W foils were cut into 2 × 2 cm samples and ultrasonically cleaned in acetone, isopropanol, methanol, and deionized water for 10 min in each solvent and then dried in an air stream [45,46]. The as-cleaned samples were contacted with a Cu spring and pressed against an O-ring in an anodization cell, which consists of a two-electrode configuration with W foil as the working electrode (anode) and the platinum foil as the counter electrode (cathode). All the experiments were performed at room temperature. The electrolytes containing various concentration of sodium fluoride (0.1, 0.2, 0.5, 0.7, and 1.0 wt.%) and sulfuric acid (0.5, 1, and 1.5 M) were used. Sets of analyses were conducted for 15, 30, 45, 60, 90, and 120 min during anodization at the voltage range of 10–50 V using a programmable power supply (MCP M10-QS1005). The as-anodized samples were raised with deionized water, dried overnight at 60 °C, and then annealed at 400 °C for 4 h with a ramping rate of 4 °C/min in the air environment.

### 2.3. Material Characterization

High-resolution scanning electron microscopy (HRSEM, JEOL, JSM-7610F) was used to analyze the morphology parameters of WO_3_ NFs. Cross-sectional images were obtained in which the samples were scrunched and measured at the angle of 30° with a tilted view to determine the thickness of the films. The crystal structures of the samples were determined from X-ray diffraction patterns recorded in the range of 2θ = 20–80° using an X-ray diffractometer (XRD, Rigaku MiniFlex 600, The Woodlands, TX, USA) with Cu Kα radiation. UV-Visible absorption spectra of the samples in the wavelength of 200–800 nm were collected using diffuse reflectance UV-Vis spectroscopy (Thermo Scientific, Waltham, MA, USA) equipped with an integrating sphere with baseline determined with barium sulfate as the reference. The photoluminescence (PL) measurements were carried out at room temperature using a photoluminescence spectrometer LS-50B (PerkinElmer, Waltham, MS, USA) equipped with a xenon discharge lamp as an excitation source and a R928 photomultiplier as a detector. The excitation radiation (300 nm) was directed on the surface of the samples at an angle of 90°. The Raman spectra were collected with a Thermo Scientific DXR Smart Raman spectrometer with a 532 nm laser as the excitation source under ambient conditions.

### 2.4. Photocatalytic Performance

The photocatalytic activity of the as-prepared WO_3_ thin films was tested by the visible light-driven degradation of toluene (200 ppm) from an air mixture, used as a model contaminant. The measurements were carried out in a flat stainless steel reactor of a volume of ca. 35 cm^3^ equipped with a quartz window, two valves, and a septum. The irradiation source, which consisted of an LED array with λ_max_ = 415 nm, was located above the sample. The as-anodized foil was placed on the bottom side and the reactor was closed with a quartz window. Subsequently, the gaseous mixture was passed through the reactor for 1 min. Then, the valves were closed and the reactor was kept in the dark for 30 min in order to achieve equilibrium. Before starting the irradiation, a reference toluene sample was taken. The concentration was determined using a gas chromatograph (TRACE 1300, Thermo Scientific), equipped with an ionization flame detector (FID) and a Phenomenex capillary column (30 mm × 25 mm, 0.5 µm). The samples (200 μL) were each dosed with a gastight syringe for 10 min. Intensity of irradiation was measured by an optical power meter and reached 15 mW/cm^2^.

### 2.5. Photoelectrochemical Activity

Photocurrent measurements were performed using an AutoLab PGSTAT 204 potentionstat–galvanostat (Methrom, Herisau, Switzerland) with the three-electrode system. Prepared samples were used as working electrodes with Ag/AgCl/0.1 M KCl and Pt mesh as the reference and counter electrodes, respectively. The active surface area of the electrode was 0.25 cm^2^. Prior to the measurement, the electrolyte 0.1 M Na_2_SO_4_ water solution was purged with argon for 1 h. Similarly, the space above the electrolyte during the measurements was purged with argon. Photocurrent measurements under UV-Vis and visible irradiation were analyzed using a 250 W Xe light source with a 420 nm cut-off filter (for visible light irradiation).

## 3. Results

To investigate the effects of the synthesis conditions and electrolyte composition (anodization potential, reaction time, and concentrations of H_2_SO_4_ and NaF) on the morphological, photoactivity, and photoelectrochemical properties of the anodic oxide WO_3_, a series of the samples were prepared. The labels of the as-prepared samples together with the synthesis conditions, morphological parameters, optical band gap, and efficiency of toluene degradation are presented in Table 1.

### 3.1. Morphology

#### 3.1.1. Effect of the Anodization Potential

**Figure 1:**
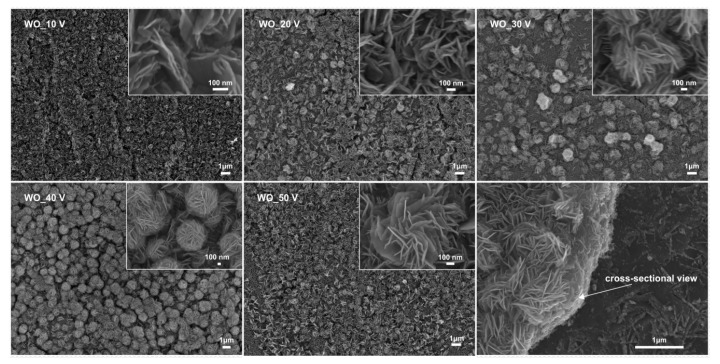
Effect of the anodization potential on the morphology of WO_3_ NFs. Fluoride content 0.5 wt.%, H_2_SO_4_ concentration 1.0 M, and anodization time 90 min.

A series of samples in the range of 10 to 50 V were synthesized to determine the effect of the anodization potential on the growth of the WO_3_ nanostructure. The results are displayed in Figure 1. Too low of an anodization potential (below 20 V) resulted in the formation of the anodic oxide layer possessing platelet nanostructure. Its irregular shape has been gradually transformed into flower-like structure when the anodization potential increased to 30 V and revealed growth of the irregular, sparsely spread flower buds with the diameter of 0.72 ± 0.1 nm. Regular shape and repeatable distribution were observed for the sample anodized at 40 V with the diameter of the flower buds equaled 1.21 ± 0.1 µm. However, a further increase to 50 V caused a decrease in the flower buds’ abundance (Figure 1). A decrease in the diameter of WO_50 V to 0.81 ± 0.2 nm was also observed. Moreover, it was found that the nanoplatelets started to deform; they were rounded and looked like developed buds. On the other hand, increasing the anodization potential resulted in an increase in the oxide layer thickness and platelet thickness from 0.1 ± 0.1 μm and 11.8 ± 0.1 nm for the WO_10 V sample to 1.2 ± 0.1 nm and 16.9 ± 0.3 nm for WO_50 V, respectively. Additionally, higher applied voltage resulted in sharpening of the edges of the nanoplatelets.

#### 3.1.2. Effect of the Anodization Time

**Figure 2:**
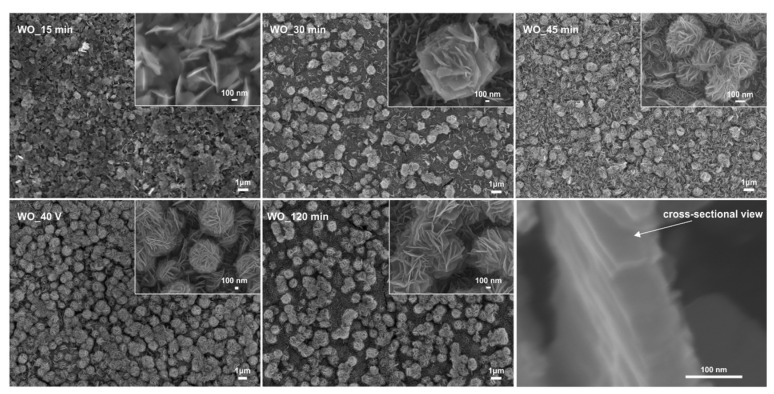
Effect of the anodization time on the morphology of the WO_3_ NFs. Fluoride content 0.5 wt.%, H_2_SO_4_ concentration 1.0 M, and anodization potential 40 V.

To investigate the effect of the anodization time on the growth of the nanoflowers, a series of experiments were conducted for different durations and the results are presented in Figure 2. The sample anodized at 40 V has been chosen to explore further its anodization time because it exhibited the most ordered, regular shape of the flower-like structure. It established that the obtained layers consisted of irregular buds when the anodization time decreased below 90 min. Moreover, in a shorter time, fewer pores were formed and an increase in heterogeneity of the layers was observed. It could be assumed that the growth of well-ordered flowers buds requires soluble species which are formed by the initial anodic growth of the oxide layer, which will be further discussed in this paper. We suggest that the continuous increase in the anodization time allows achievement of a steady state while the diameter of the flower buds, the thickness of the oxide layer, and the platelet thickness were still improved. Diameter increased to 1150 nm, oxide layer thickness increased from 0.1 ± 0.02 to 1.35 ± 0.1 μm, and platelet thickness increased from 11.2 ± 0.1 to 15.6 ± 0.2 nm, respectively for WO_15 min and WO_120 min (Table 1). It was found that the flowers buds were evenly distributed on the layer for the anodizing time of 90 min (Figure 2). However, further extension of the anodization time had a negative effect on the WO_3_ nanostructures, whereby the regular flower-like nanostructure of WO_3_ was destroyed and the anodic oxide was composed of irregular, poorly distributed flower buds. Moreover, slow transformation of the flower buds again into nanoplatelets was observed. The reason might be attributed to the higher etching rate on the WO_3_ surface layer with extension of the anodization time up to 120 min (Figure 2). Similar observation of the flower-like structure growth with an extension of the anodization time was also studied and published by the Amal group [29].

#### 3.1.3. Effect of the Sulfuric Acid Concentration

**Figure 3:**
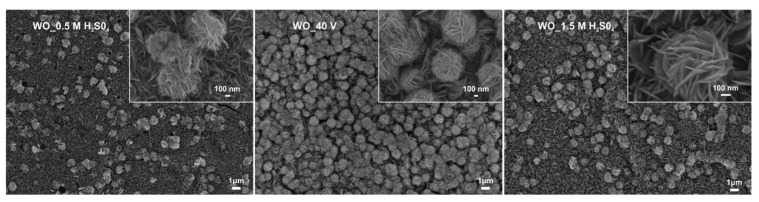
Effect of the sulfuric acid concentration on the morphology of WO_3_ NFs. Fluoride content 0.5 wt.%, anodization potential 40 V, and anodization time 90 min.

A significant impact of the sulfuric acid concentration on the homogeneity of the samples was noticed. Too low (0.5 M) or too high (1.5 M) of a concentration caused disorganization of the nanoplatelets forming the oxide layer. Moreover, for both of the mentioned concentrations, the layers consisted of evenly distributed nanoplatelets with poorly formed irregular buds. According to the literature, for the initial oxidation step occurs, the W foil needs to be in contact with the oxidizing acid, which initially oxidizes into WO_2_^2+^ and results in the formation of the continuous oxide film [29,43]. However, we assume that too low of an acid concentration resulted in too slow of a nucleation rate (WO_0.5 M H_2_SO_4_). Furthermore, it was found that the increase in the acidity of the environment resulted in etching of the WO_2_^2+^ ions, as it was confirmed from the Pourbaix diagram [47], and thus less distributed flower-buds were observed (Figure 3). El-Basiouney et al. also concluded that the dissolution of the WO_3_ oxide layer in an acidic medium takes place, which is consistent with the mechanism proposed below. The optimal concentration for forming regular flower buds was 1.0 M, reaching the highest diameter of 1.21 ± 0.1 nm. The increase of the acid concentration had a slight influence on the oxide layer and platelet thickness, the changes from 1.17 ± 0.1 to 1.0 ± 0.1 µm and from 13.4 ± 0.2 to 15.3 ± 0.3 nm, respectively for WO_0.5 M H_2_SO_4_ and WO_1.5 M H_2_SO_4_, were observed.

#### 3.1.4. Effect of the Sodium Fluoride Concentration

The effect of fluoride content concentration was investigated by the anodization of the samples at 40 V for 90 min in the electrolyte containing 0.5 M H_2_SO_4_ in the range of 0.1 to 1.0 wt.% NaF. The small content of F^−^ ions resulted in a growth of the layers consisting of the regular nanoplatelets (0.1 wt.%). The self-assembly flower buds have already occurred at 0.2 wt.% NaF content. Regularly formed buds were found for the WO_0.5 wt.% NaF sample with a diameter equal 1.21 ± 0.1 nm. Further increase of the NaF concentration led to deformations of the flower buds’ morphology to single packed nanoplatelets. Moreover, the results revealed that with the increasing amount of NaF concentration, an increase of sharpened edges of the nanoplatelets was observed. The oxide layer and platelet thickness increased slightly from 1.0 ± 0.01 to 1.15 ± 0.01 µm and from 11.3 ± 0.1 to 17.8 ± 0.3 nm, respectively for WO_0.1 wt.% NaF and WO_1.0 wt.% NaF, implying that the NaF concentration was mainly responsible for the flower bud formation.

**Scheme 1:**
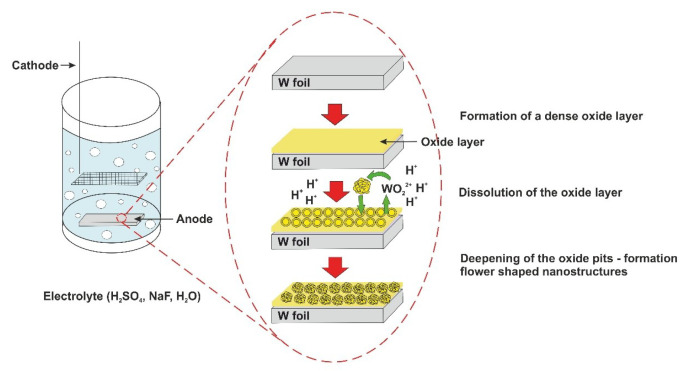
Schematic illustration of the WO_3_ NFs thin film growth process.

Similarly to the formation of the anodized titanium dioxide nanotubes films (TiO_2_ NTs) in the electrolyte containing fluorine ions [48,49], the WO_3_ NFs films were formed as a result of field-assisted oxide growth and localized chemical dissolution. Based on the above results and literature reports [28,29,50], we propose the following mechanism of the WO_3_ NFs thin film growth process: (i) formation of a dense oxide layer on W foil, (ii) activation of the barrier oxide layer by fluoride ions resulting in chemical dissolution of the oxide layer, and (iii) deepening of the oxide pits that, in time, branch out to the formation of flower-shaped nanostructures (Scheme 1). The proposed schematic illustration of the anodic growth of oxide layer is presented on Scheme 1 according to the following equations:

(1) $$W+H_{2}O\to {\mathrm{WO}}_{2}^{2+}+4H^{+}+6e^{-},$$

(2) $$2{\mathrm{WO}}_{2}^{2+}+H_{2}O\to W_{2}O_{5}+2H^{+}+2e^{-},$$

(3) $$W_{2}O_{5}+H_{2}O\to 2{\mathrm{WO}}_{3}+2H^{+}+2e^{-},$$

Reaction 1 describes the electrochemical dissolution of tungsten foil and formation of an oxide layer of WO_2_^2+^ on the surface. Subsequently, the WO_2_^2+^ ions are attracted by water molecules and form an intermediate W_2_O_5_ oxide. Since the WO_2_^2+^ and intermediate W_2_O_5_ are decomposed in an aqueous environment, the oxide layer grows. Further oxide growth is controlled by the field-enhanced ion transport through the growing oxide. This process is self-limiting under a constant applied voltage, as the field within the oxide layer is progressively reduced by the increasing oxide thickness, thus resulting in the growth of a compact WO_3_ film with finite thickness. During the initial step, disordered pits are formed and a nanoporous structure is subsequently developed by the chemical dissolution of the oxide layer or the direct complexation of WO_2_^2+^ at the oxide electrolyte interface and form soluble fluoride complexes [49]. Competition between the anodic growth of the WO_3_ oxide layer and the chemical dissolution of the tungsten oxide layer in a fluoride-containing electrolyte solution has occurred [51,52]. During the anodization process, the constant growth and chemical dissolution of the tungsten oxide layer occurs simultaneously, and a steady state is established when the growth rate at the metal–oxide interface occurs at the same as the dissolution rate of oxide film at the outer interface [13,28].

**Figure 4:**
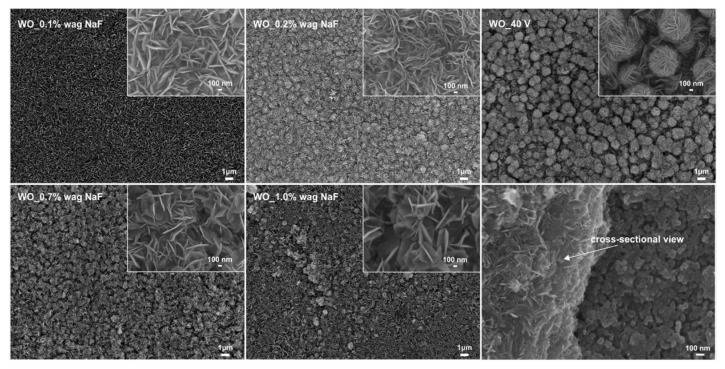
Effect of the sodium fluoride concentration on the morphology of theWO_3_ NFs. H_2_SO_4_ concentration 1.0 M, anodization potential 40 V, and anodization time 90 min.

As mentioned above, the anodic growth of the compact oxide on the metal surface and the formation of pores is governed by chemical dissolution of the formed oxide layers induced by fluoride ions from the electrolyte solution and formation of a soluble fluoride complex. The key point is to find an optimal amount of fluoride content needed to form a porous structure, allowing for the successful formation of the flower-like structure. Too low or too high of a concentration revealed presence of the nanoplatelets instead of nanoflowers (Figure 4), indicating that the presence of fluoride ions is essential to generate soluble ions (WO_2_^2+^) and form the flower-like structure. Moreover, an increase in the NaF concentration (to 1.0 wt.%) resulted in slow etching of the oxide layer and exhibited the poorly organized coexistence of flower buds with nanoplatelets (Figure 2). Lai [53] also investigated the influence of the fluoride content on the growth of anodic WO_3_ nanotubular structure and realized that optimization of fluoride ions played a crucial role in controlling formation of the chemical dissolution reaction on the interface of W/WO_3_, and thus growth of the nanotubes. It should be remarked that dissolution of WO_3_ occurs over the entire W foil; thus, with extending oxidation time, we observed gradual transformation of the nanoplatelets into a flower-like structure after 90 min of the anodization process. Further time extension resulted in opposite effects (Figure 2). Moreover, a literature survey reports that the dissolution of WO_3_ in acid medium takes places via the formation of WO_2_^2+^ species [54]. Therefore, it might lead to the precipitation of primary formed WO_2_^2+^ and allows for thickening of the WO_3_ nanostructured film. However, we observed that the increase of the acid concentration resulted in the formation of a slightly thinner oxide layer (Table 1). Therefore, we assume that the dissolution of the oxide layer could be driven by the instability of WO_2_^2+^ in the presence of aqueous electrolyte solution at room temperature and presence of high anodization potential.

### 3.2. Crystallographic Structure

**Figure 5:**
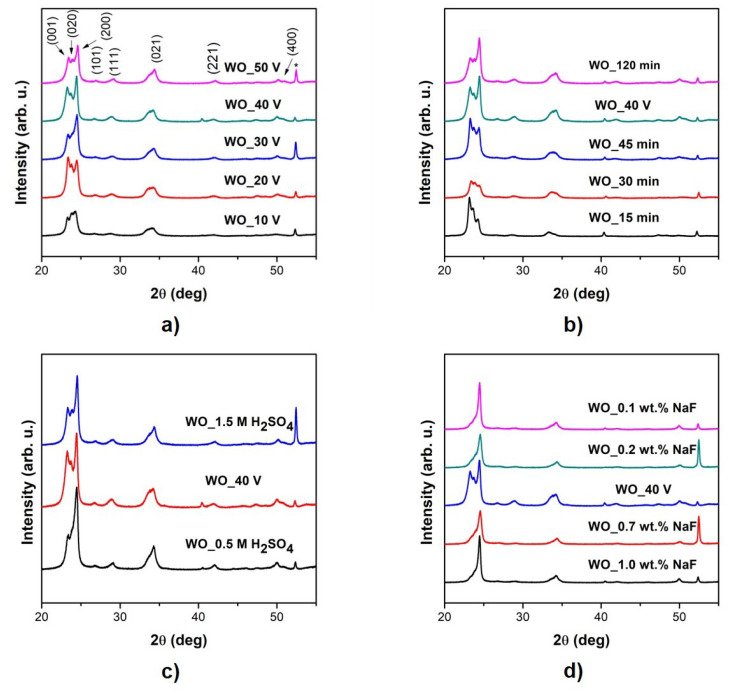
XRD patterns of the WO_3_ NFs samples differing in (**a**) applied potential, (**b**) anodization time, (**c**) H_2_SO_4_, and (**d**) NaF concentration.

Figure 5 displays the X-ray patterns of the self-assembled WO_3_ NFs film. The reflection patterns of the WO_3_ could be indexed to a triclinic phase. The WO_3_ phase was represented by the peaks (001), (020), (200), (101), (111), (021), (221), and (400) crystal planes at 2θ located around 23.3°, 23.8°, 24.4°, 26.8°, 28.9°, 33.8°, 41.4°, and 50.5°. The peaks indexed to W foils were found at 2θ of approximately 52.5 (denoted in Figure 5 “*”), 58.5, and 73.4. The XRD patterns of the samples anodized for 90 min in the potential range of 10–50 V are given in Figure 5a. As noted, the potential of 10 V was too low for successful formation of the oxide layers; thus, it was hard to establish any of the peaks in range of 23–25°. It was found that with increasing anodization potential, the intensity of the peak (200), characteristic for triclinic phase of WO_3_, increased. However, further rise to 50 V resulted in the intensity decrease. As the anodization time increased, we observed that the intensity of the (001) peak decreased, whereas the (200) one started to intensively rise and reached its highest intensity with an oxidation time of 120 min (Figure 5b). As the sulfuric acid concentration increased, the intensity of the (200) peak started to decrease, whereas the intensity of peaks indexed to (001) and (020) became higher (Figure 5c). An optimum concentration which allowed for the growth of the oxide layer was 1.0 M. Interestingly, concentration of fluoride content strongly affected the peaks’ heights indexed to WO_3_ (Figure 5d). Only the WO_0.5 wt.% NaF sample revealed the presence of the peaks (001), (020), (200), (101), and (111), whereas the rest of the samples in this series possessed the intensive peak indexed to (200). Reduction of the (001) peak, and thus enhancement of the (200) peak intensity in all the samples, could be ascribed to the improvement of the nanostructured layer and compact oxide layers obtained at different conditions. The WO_3_ NFs samples possessed analogous crystallinity, while different synthesis conditions resulted in changes of the refined lattice parameters a, b, and c as well as unit cell volume, which are gathered in Table S1.

**Figure 6:**
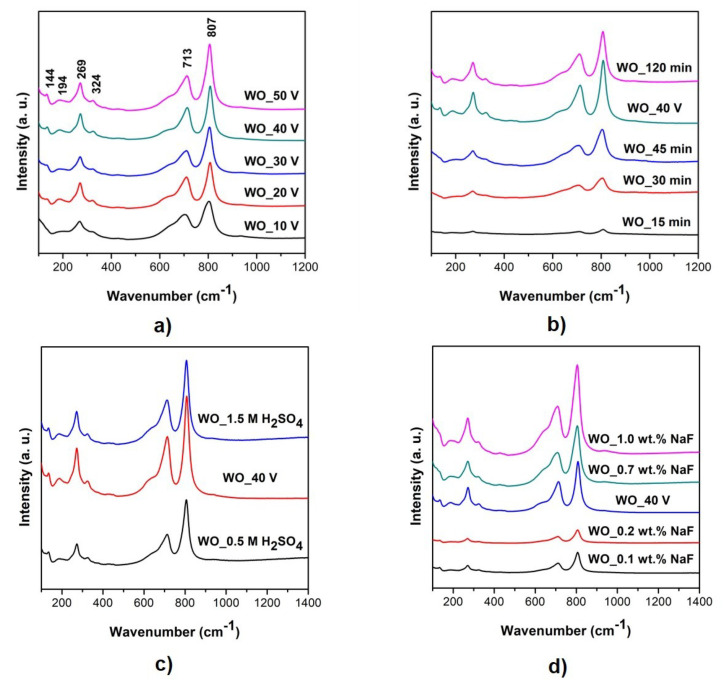
Raman spectra of the WO_3_ NFs samples differing in (**a**) anodization potential, (**b**) anodization time (**c**) H_2_SO_4_, and (**d**) NaF concentration.

The Raman spectra of the samples are displayed in Figure 6. As it can be seen, we can determine three frequency regions. The first one appeared at lower frequencies (below 200 cm^−1^) in regard to the relative translational or rotational motions of the WO_6_ octahedral. The second region occurs between wave numbers of 200–400 cm^−1^, indicating the O-W-O bending mode. The last region with peaks located at around 600–900 cm^−1^ is indexed to the O-W-O stretching modes [55]. The Raman spectra confirmed the WO_3_ triclinic phase due to the presence of the characteristic peaks at around 144, 194, 269, 324, 713, and 807 cm^−1^. No Raman signal corresponding to the tungsten was observed. The bands of 269 and 324 cm^−1^ can be attributed to the δ bending (O-W-O) and ν (W-O-W) vibrations modes of the bridging oxygen [56]. The strongest peaks observed at 713 and 807 cm^−1^ can be assigned to the stretching modes arising from O-W-O [23,56]. All the samples exhibited the same features, but as the preparation conditions changed, an intensity of the peaks starts to arise (especially at around 269 and 807 cm^−1^), indicating the formation of the oxide layer (Figure 6a–d).

### 3.3. Optical and Photoluminescence Properties

**Figure 7:**
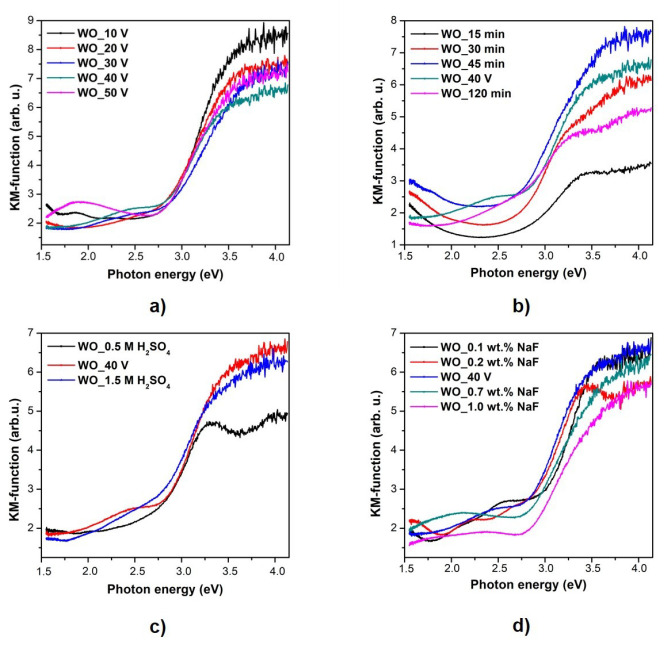
Optical band gap spectra of the WO_3_ NFs samples differing in (**a**) anodization potential, (**b**) anodization time, (**c**) H_2_SO_4_, and (**d**) NaF concentration.

The change in the optical properties of the as-prepared WO_3_ films by the photoabsorption studies (Figure S1) and corresponding optical band gap energy as presented in Figure 7 were investigated. The absorption edge of the series difference in the applied potential was located approximately of 470 nm for all samples (Figure S1a). As the anodization time increase from 15 to 120 min, the absorption edge shifted from 460 to 540 nm (Figure S1b), respectively. Higher acid concentration practically did not influence on the absorption edge, which was around 475 nm (Figure S1c). For the series differing in fluoride ions concentration, the absorption edge was around 450 nm for all samples except the one obtained with the lowest amount of NaF (0.1 wt.%)—approximately 430 nm (Figure S1d). An absorption shift in the 450–800 nm range was observed for all samples, confirming changes in the structures of the obtained samples. Raised absorbance values at higher wavelengths could be attributed to the presence of oxygen vacancies on the surface film. Furthermore, it was found that as the anodization time increased, the photoabsorbance values decreased due to a decrease in the amount of suboxides and oxygen vacancies. A similar observation was described by Amal et al., who analyzed the influence of duration time period on the flower-shaped WO_3_ growth [29].

The band gap of WO_3_ NFs films can be determined by considering the indirect transition between 2p electrons from the valence band (VB) of oxygen and the 5d the conduction band (CB) of tungsten based on the Tauc’s plot according to the following equation [57,58]:

(4) $$\alpha h\nu \;=\;A{\left(h\nu -E_{g}\right)}^{n},$$

where α, ν, A, and E_g_ are absorption coefficient, light frequency, proportionality constant, and band gap, respectively. The band gap energies of the WO_3_ NFs samples were calculated with Equation (4), and the data were collected in Table 1 and displayed in Figure 7. The values were in accordance with the literature, where the band gap values for the flower-shaped WO_3_ film were around 2.5–2.75 eV [37,40].

**Figure 8:**
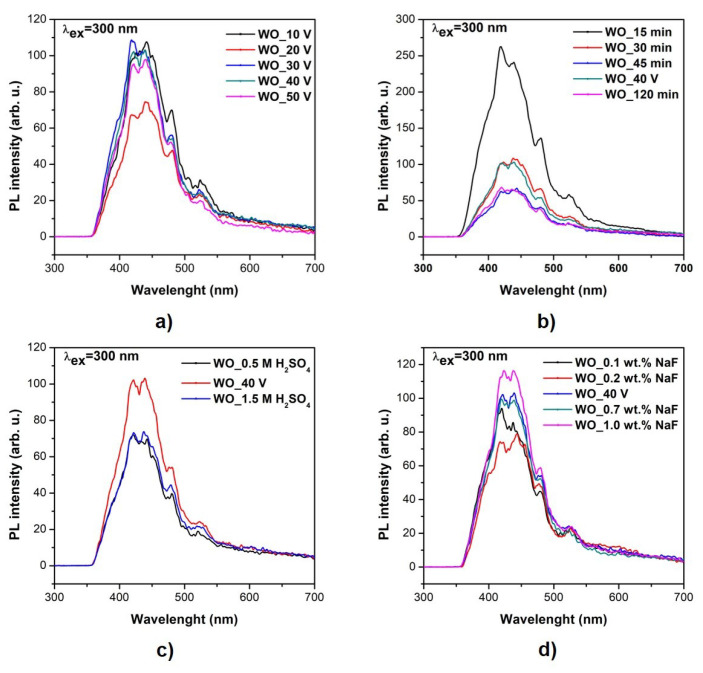
Photoluminescence spectra of the WO_3_ NFs samples differing in (**a**) anodization potential, (**b**) anodization time, (**c**) H_2_SO_4_, and (**d**) NaF concentration.

Photoluminescence spectra for the as-obtained WO_3_ NFs samples in four different series are shown in Figure 8a–d. Typically for the oxygen metal-based semiconductors, the PL spectra are composed of UV emission and a visible emission band attributed to the surface defects. All the samples exhibited the same PL features with different intensity depending on the preparation conditions. The emission was positioned at around 420, 438, 481, and 527 nm. The values at around 420, 438, and 481 nm might be attributed to the presence of intrinsic defects such as oxygen vacancies, giving rise to donor states located below the CB. The emission located at wavelength of 527 nm indicated the possibility of band recombination (intrinsic states rather than surface states). The values are consistent with those reported for the others semiconductors, such as TiO_2_ and ZnO [59,60].

### 3.4. Photocatalytic Activity

**Figure 9:**
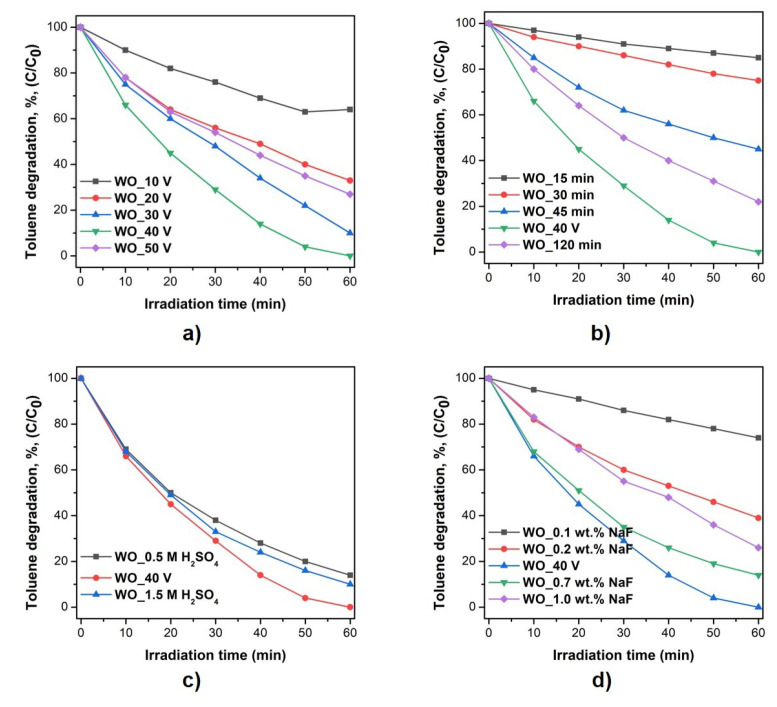
Photoactivity of the WO_3_ NFs samples in gas phase degradation of toluene under Vis light irradiation (λ_max_ = 415 nm) differing in (**a**) anodization potential, (**b**) anodization time, (**c**) H_2_SO_4_, and (**d**) NaF concentration.

The potential environmental applications of the as-prepared WO_3_ samples were investigated in a model reaction of toluene degradation from the air mixture to simple degradation products like CO_2_ and H_2_O. This approach was employed to analyze the effect of the preparation conditions, anodization potential, and time, as well as electrolyte composition, concentration of sulfuric acid, and sodium fluoride, on the WO_3_ NFs photoactivity. The obtained results are displayed in Figure 9 and Table 1. It was found that samples preparation route by changing applied potential, anodization time, and fluoride content significantly influenced the photocatalytic activity. In the case of different H_2_SO_4_ concentration, the samples WO_0.5 M H_2_SO_4_, WO_1.0 M H_2_SO_4_, and WO_1.5 M H_2_SO_4_ had comparable efficiencies of 86%, 100%, and 90%, respectively. As shown in Figure 9a–d, the highest photocatalytic activity, reaching 100% of toluene removal after 60 min of irradiation, achieved the sample prepared in the following conditions: anodization potential 40 V, anodization time 90 min, fluoride content 0.5 wt.%, and H_2_SO_4_ concentration 0.5 M. Further increases of each of the parameters resulted in an opposite effect; a decrease in photoactivity was observed for WO_50 V, WO_120 min, WO_1.5 M H_2_SO_4_, and WO_0.1 wt.% NaF—73%, 78%, 90%, and 74%, respectively.

**Figure 10:**
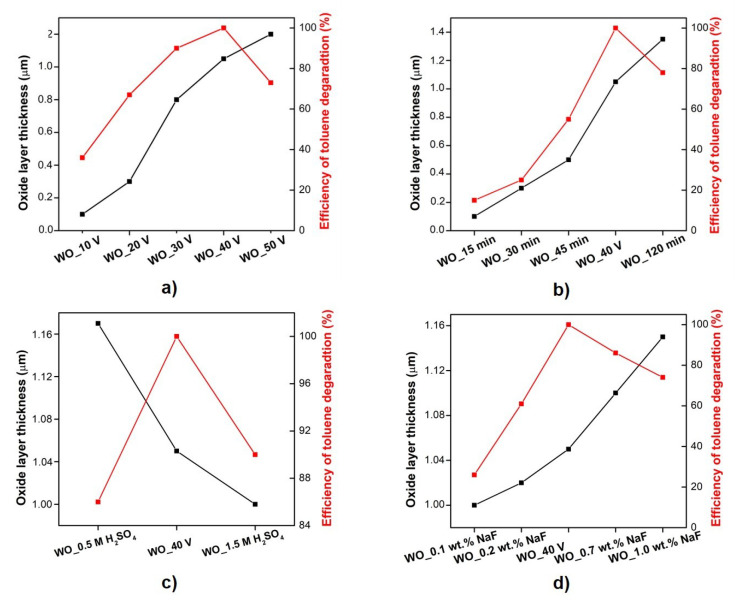
Influence of the oxide layer thickness on the efficiency of toluene degradation in the series differing in (**a**) applied potential, (**b**) anodization time, (**c**) H_2_SO_4_, and (**d**) NaF concentration.

In line with the other photocatalysts, the photoactivity of WO_3_ depends on the lifetime of photogenerated charge carriers. Surface electron–hole recombination is extremely high with most of charge carriers recombining on the photocatalyst surface before the redox reactions, and this recombination rate must be reduced in order to improve the photocatalytic activity. It seems that a simple toluene degradation route in presence of the WO_3_ samples relies on the attack of the OH• radical to the methyl group of toluene. The key parameter that influence the improvement of the photocatalytic activity toward air pollutant degradation is morphology control. According to the literature, it was found that the flower-like three-dimensional (3D) structure photocatalysts possesses (i) a larger surface area and (ii) plenty of mesopores with ordered open-pore frameworks, into which photocatalysts may effectively harvest visible light due to multiple scattering [61,62]. Moreover, the large surface of the flower-like structure could decrease the recombination efficiency of the photoexcited carries and favor their transfer to the surface to react with organic pollutants. When comparing the photoactivity of the as-prepared WO_3_ samples with their morphological parameters, it was obvious that the well-ordered, regular shape of nanoflower array films was more efficient than the randomly occurring flower buds or nanoplatelets (Table 1, Figure 10). It could be ascribed to the more effective separation for the photogenerated electron–hole pairs and the larger surface area of the nanoflower structure [63]. As apparent from the above discussed experimental data, the highest photocatalytic activity in the reaction of toluene decomposition (100%) after 60 min of LED irradiation (λ_max_ = 415 nm) was reached for the WO_40 V sample prepared in the following conditions: 40 V, 90 min, 1.0 M H_2_SO_4_, and 0.5 wt.% NaF. This sample also possessed the most uniform distribution and regular shape of the flower buds with a diameter equal to 1.21 ± 0.1 µm. We noticed that the efficiency of toluene degradation increased with increasing thickness of WO_3_ array film, and then had a downward trend (Figure 10a–d). The experimental results indicated that the 1.05 μm thick WO_3_ film appeared with a maximum photodegradation efficiency of the toluene removal. Previous literature suggests that if the metal oxide thin film is thicker than the depth of light penetration, the bottom film absorbs only a few incident photons and serves as an inter-support layer, and resulted in the decrease of the photocatalytic activity for a thick film [64].

### 3.5. Photoelectrochemical Activity

**Figure 11:**
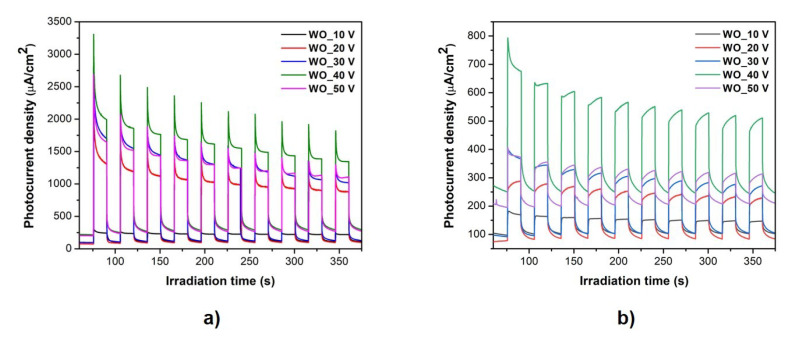
Photocurrent response of selected WO_3_ samples under (**a**) UV-Vis and (**b**) Vis irradiation (λ > 420 nm) at 1.5 V in 0.1 M Na_2_SO_4_. The switch on/off period is 30 s.

Photoelectrochemical response of the samples prepared at different applied potential was evaluated as photocurrent measurements in light off/on cycles with 1.5 V applied voltage. Photocurrent was registered under both UV-Vis (Figure 11a) and Vis irradiation (Figure 11b). No significant decay of the photocurrent was observed during the photoelectrochemical measurements under both sources of irradiation, indicating good stability of obtained photoelectrodes. The photocurrent under UV-Vis light was approximately five times higher in comparison with visible light irradiation. In addition, the same tendency in sample photoelectroactivity was observed for both types of light sources; namely, as the anodizing voltage increased to 40 V, the measured photocurrent increased and then decreased (for a sample obtained at 50 V). This could be related to the high ordering of the platelet structure and the improvement of the nanostructured layer thickness with larger surface area which was achieving with increasing anodization voltage (up to 40 V). Prepared samples, especially the most photoactive sample (WO_40 V), exhibited the large surface of the flower-like structure, which increased the interface between the oxide layer and electrolyte, facilitating incident photons absorbance and the charge transfer for the separation of photoexcited electron–hole pairs.

## 4. Conclusions

In summary, we have described a simple and environmental friendly one-step anodization synthesis method of the WO_3_ NFs thin films in the aqueous solution containing fluoride ions. The effects of synthesis conditions, applied potential, and anodization time, as well as electrolyte composition, concentration of H_2_SO_4_, and NaF contents, on the morphology, photocatalytic activity, and photoelectrochemistry properties of the WO_3_ photocatalysts was presented. The results revealed the growth of the nanoplatelets which were slowly transformed into the nanoflower structure depending on the synthesis parameters. The optimal conditions allowing the synthesis of the well-organized, regularly shaped flower buds with a diameter of 1.21 nm were: anodization potential of 40 V, anodization time 90 min, the electrolyte containing 1.0 M H_2_SO_4_, and 0.5 wt.% NaF. The photocatalytic activity of the as-prepared WO_3_ samples toward decomposition of air contaminants was investigated in the model reaction of toluene degradation. Moreover, we found that the morphology control was the key avenue to improve the photocatalytic activity. Hence, the samples which consisted of the irregularly shaped nanoplatelets exhibited lower photocatalytic activity than those with the flower-like structure. The highest photoactivity in toluene degradation and in photocurrent generation under UV-Vis and Vis irradiation achieved the sample with the most ordered and regular flower buds, WO_40 V. Moreover, we noticed that the thickness of the oxide layer was directly related with the photocatalytic efficiency; the thicker the WO_3_ layer was, the higher the decline in the photoactivity was observed because of difficulties of light penetration into deeper layers, although the highest photoactivity was exhibited for the sample with thickness of 1.05 μm (100%, WO_40 V), and a further increase of the oxide layer thickness resulted in an opposite effect. These finding suggest that a self-assembling, regularly shaped flower-like WO_3_ thin film activated with low-powered LEDs as an irradiation source (λ_max_ = 415 nm) could be a promising material for air purification. The synthesis of visible light active and stable photocatalysts can boost the technology of air purification since they exploit a renewable energy source and allow avoidance of extra operational costs and other limitations associated with artificial illumination.

## Figures and Tables

**Table 1:** Sample label, morphology parameters, optical band gap, and efficiency of toluene degradation of the obtained samples.

Sample Label	Preparation Conditions (Electrolyte Composition and Anodization Parameters)		Flower Diameter (nm)	Oxide Layer Thickness (µm)	Platelets Thickness (nm)	Optical Band Gap (eV)	Efficiency of Toluene Degradation after 60 min of Irradiation (%)
	Constant Parameters	Variable Parameters					
WO_10 V	1.0 M H_2_SO_4_, 0.5 wt.% NaF, 90 min	10 V	- **	0.1 ± 0.1	11.8 ± 0.1	2.75	36
WO_20 V	1.0 M H_2_SO_4_, 0.5 wt.% NaF, 90 min	20 V	- **	0.3 ± 0.1	12.8 ± 0.2	2.61	67
WO_30 V	1.0 M H_2_SO_4_, 0.5 wt.% NaF, 90 min	30 V	0.72 ± 0.1	0.8 ± 0.1	13.6 ± 0.2	2.65	90
WO_40 V *	1.0 M H_2_SO_4_, 0.5 wt.% NaF, 90 min	40 V	1.21 ± 0.1	1.05 ± 0.1	14.9 ± 0.3	2.52	100
WO_50 V	1.0 M H_2_SO_4_, 0.5 wt.% NaF, 90 min	50 V	0.81 ± 0.2	1.2 ± 0.1	16.9 ± 0.3	2.65	73
WO_15 min	1.0 M H_2_SO_4_, 0.5 wt.% NaF, 40 V	15 min	- **	0.1 ± 0.02	11.2 ± 0.1	2.66	15
WO_30 min	1.0 M H_2_SO_4_, 0.5 wt.% NaF, 40 V	30 min	0.91 ± 0.1	0.3 ± 0.05	12.3 ± 0.1	2.64	25
WO_45 min	1.0 M H_2_SO_4_, 0.5 wt.% NaF, 40 V	45 min	1.04 ± 0.2	0.5 ± 0.1	13.1 ± 0.2	2.56	55
WO_40 V *	1.0 M H_2_SO_4_, 0.5 wt.% NaF, 40 V,	90 min	1.21 ± 0.1	1.05 ± 0.2	14.9 ± 0.2	2.52	100
WO_120 min	1.0 M H_2_SO_4_, 0.5 wt.% NaF, 40 V	120 min	1.52 ± 0.2	1.35 ± 0.1	15.6 ± 0.2	2.40	78
WO_0.5 M H_2_SO_4_	0.5 wt.% NaF, 40 V, 90 mi	0.5 M H_2_SO_4_	1.15 ± 0.1	1.17 ± 0.1	13.4 ± 0.2	2.63	86
WO_40 V *	0.5 wt.% NaF, 40 V, 90 min	1.0 M H_2_SO_4_	1.21 ± 0.1	1.05 ± 0.1	14.9 ± 0.2	2.52	100
WO_1.5 M H_2_SO_4_	0.5 wt.% NaF, 40 V, 90 min	1.5 M H_2_SO_4_	1.10 ± 0.1	1.0 ± 0.1	15.3 ± 0.3	2.55	90
WO_0.1 wt.% NaF	1.0 M H_2_SO_4_, 40 V, 90 min	0.1 wt.% NaF	- **	1.0 ± 0.01	11.3 ± 0.1	2.79	26
WO_0.2 wt.% NaF	1.0 M H_2_SO_4_, 40 V, 90 min	0.2 wt.% NaF	- **	1.02 ± 0.1	12.1 ± 0.2	2.62	61
WO_40 V *	1.0 M H_2_SO_4_, 40 V, 90 min	0.5 wt.% NaF	1.21 ± 0.1	1.05 ± 0.01	14.9 ± 0.3	2.52	100
WO_0.7 wt.% NaF	1.0 M H_2_SO_4_, 40 V, 90 min	0.7 wt.% NaF	- **	1.1 ± 0.01	15.9 ± 0.3	2.55	86
WO_1.0 wt.% NaF	1.0 M H_2_SO_4_, 40 V, 90 min	1.0 wt.% NaF	- **	1.15 ± 0.01	17.8 ± 0.3	2.66	74

* The WO_40 V sample was synthesized in the following conditions: 90 min, 40 V, 0.5 wt.% NaF, and 1.0 M H_2_SO_4_ in each series to compare the influence of preparation conditions. ** The formation of the flower buds was not observed.

## Supplementary Materials

The following are available online at https://www.mdpi.com/1996-1944/13/16/3506/s1, Figure S1: Photoabsorption spectra of the WO_3_ NFs samples from four series each with a different (a) applied potential, (b) anodization time, (c) H_2_SO_4_, and (d) NaF concentration., Table S1: Lattice parameters of the WO_3_ NFs samples.
