# Supplementary material for: Enhanced Visible Light Active WO3 Thin Films Toward Air Purification: Effect of the Synthesis Conditions
Source: Materials (Basel). 2020 Aug 8;13(16):3506. doi: 10.3390/ma13163506 (PMC7475911; doi:10.3390/ma13163506)
Supplement: Supplementary file 1 [file materials-13-03506-s001.pdf]

materials

MDPI

Supplementary data

Enhanced visible-light active WO_3 thin films towards air purification: effect of the synthesis conditions

Anna Pancielejko [1], Marta Rzepnikowska [2], Adriana Zaleska-Medynska [2], Justyna Łuczak [1], Paweł Mazierski [2*]

[1] Department of Engineering Process and Chemical Technology, Faculty of Chemistry, Gdansk University of Technology, 80-233 Gdansk, Poland; anna.pancielejko@pg.edu.pl (A.P.); justyna.luczak@pg.edu.pl (J.Ł.)

[2] Department of Environmental Technology, Faculty of Chemistry, University of Gdansk, 80-308 Gdansk, Poland; marrzepn21@gmail.com (M.R.); adriana.zaleska-medynska@ug.edu.pl (A.Z.-M.)

* Correspondence: pawel.mazierski@ug.edu.pl; Tel.: +48-58-523-52-30

Received: 20 July 2020; Accepted: 06 August 2020; Published: date

Table S1. Lattice parameters of the WO_3 NFs samples.

Sample label	a (Å)	b (Å)	c (Å)	V (Å³)
WO_15 min	7.3454(8)	7.5472(8)	3.8543(6)	213.67(9)
WO_30 min	7.2786(1)	7.4696(5)	3.8830(1)	211.11(8)
WO_45 min	7.2858(7)	7.4860(4)	3.8220(3)	208.51(9)
WO_40 V	7.2782(9)	7.4782(4)	3.8191(1)	207.88(7)
WO_120 min	7.2668(1)	7.4664(4)	3.8130(8)	206.88(7)
WO_10 V	7.3065(8)	7.5073(2)	3.8339)5)	210.30(3)
WO_20 V	7.2740(1)	7.4633(9)	3.8064(8)	205.79(9)
WO_30 V	7.2549(7)	7.4542(8)	3.8068(6)	205.87(7)
WO_40 V	7.2782(9)	7.4782(4)	3.8191(1)	207.88(7)
WO_50 V	7.2310(2)	7.4296(7)	3.7942(9)	203.84(5)
WO_0.1 wt.% NaF	7.2684(4)	7.4681(3)	3.8139(3)	207.02(7)
WO_0.2 wt.% NaF	7.2313(7)	7.4300(3)	3.7944(8)	203.87(5)
WO_40 V	7.2782(9)	7.4782(4)	3.8191(1)	207.88(7)
WO_0.7 wt.% NaF	7.2511(0)	7.4503(1)	3.8048(4)	205.54(9)
WO_1.0 wt.% NaF	7.2376(9)	7.4365(3)	3.7977(9)	204.41(1)
WO_0.5 M H_2SO_4	7.2445(4)	7.4436(6)	3.8013(9)	204.99(1)
WO_40 V	7.2782(9)	7.4782(4)	3.8191(1)	207.88(7)
WO_1.5 M H_2SO_4	7.2309(6)	7.4296(1)	3.7942(7)	203.83(9)

* The WO_40 V sample was synthesised in the following conditions: 90 min, 40 V, 0.5 wt.% NaF and 1.0 M H_2SO_4 in each series to compare the influence of preparation conditions.

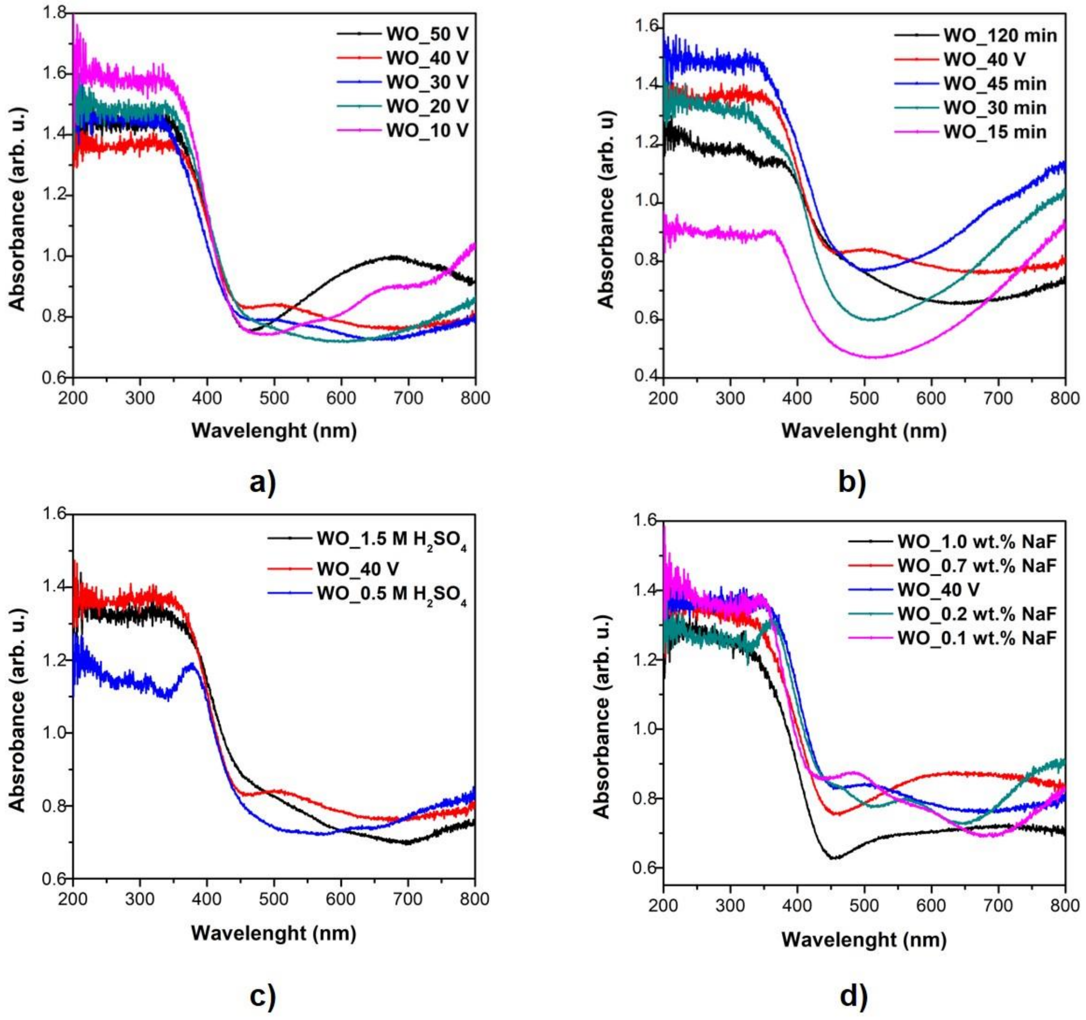

Figure S1. Photoabsorption spectra of the WO_3 NFs samples from four series each with a different (a) applied potential, (b) anodization time, (c) H_2SO_4 and (d) NaF concentration.
